# MMP-3 Knockout Induces Global Transcriptional Changes and Reduces Cerebral Infarction in Both Male and Female Models of Ischemic Stroke

**DOI:** 10.3390/ijms25137383

**Published:** 2024-07-05

**Authors:** Milton H. Hamblin, Austin C. Boese, Rabi Murad, Jean-Pyo Lee

**Affiliations:** 1 Division of Biomedical Sciences, School of Medicine, University of California, Riverside, Riverside, CA 92521, USA; 2 Health Sciences Center, Tulane University, New Orleans, LA 70112, USA; 3 School of Medicine, Emory University, Atlanta, GA 30322, USA; austin.c.boese@emory.edu; 4 Bioinformatics, Sanford Burnham Prebys Medical Discovery Institute, La Jolla, CA 92037, USA; rmurad@sbpdiscovery.org; 5 Department of Physiology, Tulane University School of Medicine, New Orleans, LA 70112, USA

**Keywords:** inflammation, matrix metalloproteinase-3, RNA sequencing, ingenuity pathway analysis, gene set enrichment analysis, stroke, transcriptome

## Abstract

Ischemic stroke followed by reperfusion (IR) leads to extensive cerebrovascular injury characterized by neuroinflammation and brain cell death. Inhibition of matrix metalloproteinase-3 (MMP-3) emerges as a promising therapeutic approach to mitigate IR-induced stroke injury. We employed middle cerebral artery occlusion with subsequent reperfusion (MCAO/R) to model ischemic stroke in adult mice. Specifically, we investigated the impact of MMP-3 knockout (KO) on stroke pathophysiology using RNA sequencing (RNA-seq) of stroke brains harvested 48 h post-MCAO. MMP-3 KO significantly reduced brain infarct size following stroke. Notably, RNA-seq analysis showed that MMP-3 KO altered expression of 333 genes (252 downregulated) in male stroke brains and 3768 genes (889 downregulated) in female stroke brains. Functional pathway analysis revealed that inflammation, integrin cell surface signaling, endothelial- and epithelial-mesenchymal transition (EndMT/EMT), and apoptosis gene signatures were decreased in MMP-3 KO stroke brains. Intriguingly, MMP-3 KO downregulated gene signatures more profoundly in females than in males, as indicated by greater negative enrichment scores. Our study underscores MMP-3 inhibition as a promising therapeutic strategy, impacting multiple cellular pathways following stroke.

## 1. Introduction

Stroke is a leading cause of death and disability in the United States [1]. Aging is a major risk factor for cerebrovascular diseases, and about 75% of strokes afflict individuals 65 years of age or older [2]. Approximately 87% of strokes are classified as ischemic [1] and occur due to thrombosis in cerebral arteries. Clinically, ischemia followed by reperfusion (IR) results in extensive cerebrovascular injury and neurological dysfunction [3,4]. Thrombolysis by tissue plasminogen activator (tPA) [5] and thrombectomy [6,7] are current primary treatments for acute ischemic stroke and have serious limitations. For instance, tPA, the only thrombolytic agent approved by the U.S. Food and Drug Administration, has a narrow therapeutic window of 4.5 hours (h) after stroke onset [8] and increases the chances of hemorrhagic transformation (HT) [9,10]. Thus, new drug targets for ischemic stroke are greatly needed. 

Maintaining blood-brain barrier (BBB) integrity is critical for brain function and homeostasis [11,12,13,14]. Ischemic stroke compromises BBB integrity, which leads to further brain injury even after the initial ischemic insult [15,16]. Preclinical studies reveal that ischemia-reperfusion causes a biphasic BBB opening [17]; an initial but reversible opening occurs several hours post-reperfusion [18], which is then followed by a later irreversible opening that exacerbates brain damage [19]. BBB injury results in part from inflammation and upregulation of matrix metalloproteinases (MMPs) [15,16,20,21,22,23,24,25]. Thus, ameliorating BBB damage is a promising therapeutic strategy for stroke. MMP-3 (stromelysin-1) is a 51-kDa protein [26] that degrades components of the extracellular matrix (ECM) and has important roles in tissue remodeling and wound healing [27]. As one of the major inducible MMPs, MMP-3 can also activate latent pro-MMP-9 [28,29,30]. MMP-3 is upregulated within several hours after stroke and this corresponds with the initial opening of the BBB [31]. MMP-3 deficient mice exhibit reduced tPA-induced HT after stroke [31], and MMP-3 exacerbates HT in hyperglycemic rats [32]. We previously reported significantly increased MMP-3 levels in the ipsilesional hemisphere of mouse brains at 48 h post-stroke [33]. We also found that human neural stem cell (hNSC) transplantation reduced MMP-3 levels in aged mouse stroke brains [33]. However, the molecular mechanisms through which MMP-3 contributes to subacute stroke IR injury remain poorly understood.

In this study, we assessed the effects of MMP-3 genetic knockout (MMP-3 KO) on infarct volume and gene expression in the brains of mice following middle cerebral artery occlusion with subsequent reperfusion (MCAO/R). Our RNA-seq analysis revealed significant downregulation of gene expression signatures for neuroinflammation, endothelial- and epithelial-mesenchymal transition (EndMT/EMT), and apoptosis in MMP-3 KO mouse stroke brains compared to MMP-3 wild-type (WT) controls. Stratification of gene expression by sex revealed depletion of similar gene signatures in both males and females, but to a greater extent in females. Our study is the first to utilize RNA-seq to identify altered gene signatures and pathways associated with improved subacute stroke phase outcome in MMP-3 KO mice.

## 2. Results

### 2.1. Genetic Knockout of MMP-3 Reduces Infarct Volume in Stroke Mouse Brains

**Figure 1:**
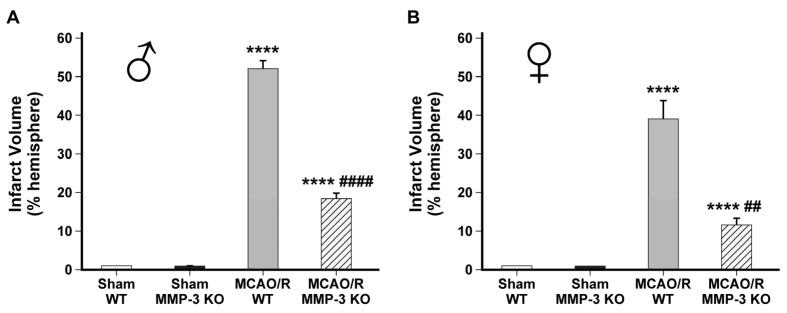
MMP-3 knockout reduces infarct volume in mice after ischemic stroke. (**A**) Infarct volume calculated by TTC staining of male mouse brains harvested 48 h post-MCAO (MMP-3 KO vs. WT). **** *p* < 0.0001 vs. sham; #### *p* < 0.0001 vs. MCAO/R WT. (n = 6, sham WT; n = 6, sham MMP-3 KO; n = 5, MCAO/R WT; n = 5, MCAO/R MMP-3 KO). (**B**) Infarct volume calculated by TTC staining of female mouse brains harvested 48 h post-MCAO (MMP-3 KO vs. WT). **** *p* < 0.0001 vs. sham; ## *p* < 0.01 vs. MCAO/R WT. (n = 6, sham WT; n = 6, sham MMP-3 KO; n = 5, MCAO/R WT; n = 5, MCAO/R MMP-3 KO). Data are presented as mean ± SEM. MCAO/R, middle cerebral artery occlusion with reperfusion.

We assessed the effects of MMP-3 genetic deletion on infarct volume in stroke brains. We measured ischemic lesion volume 48 h post-MCAO by staining brain sections with TTC. Compared to brains from MCAO/R WT mice, brains from MMP-3 KO mice had significantly smaller infarct sizes measured at 48 h post-stroke. The mean infarct volume of the ipsilesional hemisphere in male WT stroke brains was 52.02 ± 2.10% (**** *p* < 0.0001 vs. sham). In male MMP-3 KO stroke mice, the mean infarct volume was 18.27 ± 1.56% (**** *p* < 0.0001 vs. sham), which was significantly smaller than in the male WT MCAO/R group (^####^ *p* < 0.0001 vs. MCAO/R WT) (Figure 1A). The mean infarct volume of the ipsilesional hemisphere in female WT stroke brains was 38.99 ± 4.59% (**** *p* < 0.0001 vs. sham). In female MMP-3 KO stroke mice, the mean infarct volume was 11.55 ± 1.62% (**** *p* < 0.0001 vs. sham), which was significantly smaller than in the female WT MCAO/R group (^##^ *p* < 0.01 vs. MCAO/R WT) (Figure 1B). These results indicate that genetic deletion of MMP-3 attenuates brain tissue loss from ischemic stroke.

### 2.2. MMP-3 Deletion Induces Global Transcriptional Changes in the Brains of Male and Female Mice in the Subacute Stroke Phase

**Figure 2:**
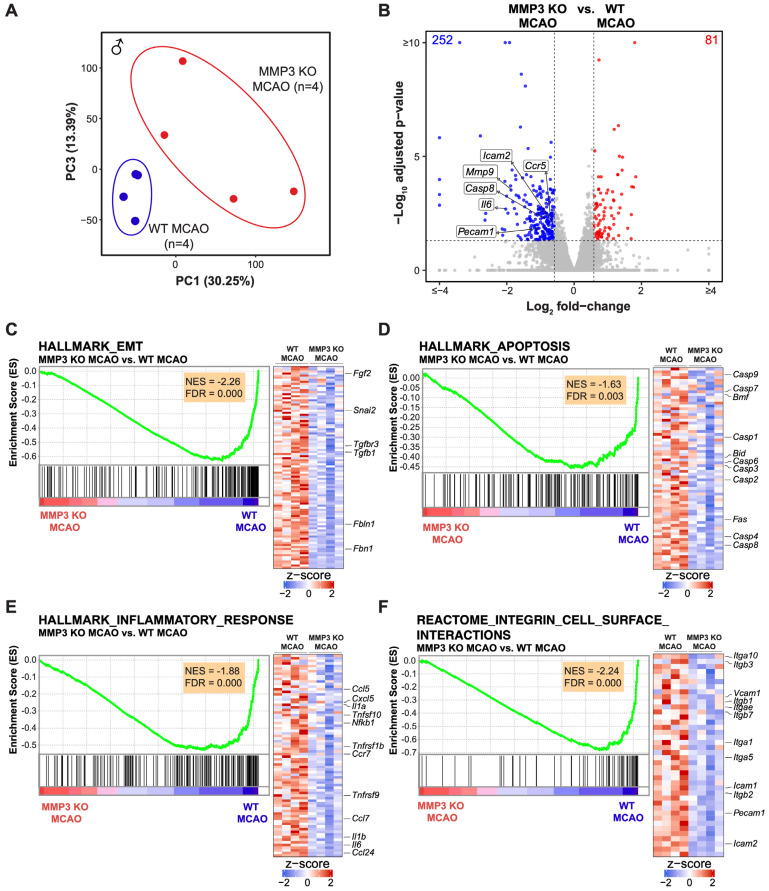
MMP-3 KO induces global transcriptional changes in the brains of male mice by 48 h post-stroke. (**A**) Principal component analysis (PCA) of transcriptomes from whole-brain tissue of male MMP-3 KO (n = 4, red) and male WT (n = 4, blue) mouse brains harvested at 48 h post-stroke. Each dot represents a biological replicate of an RNA-seq sample, and principal component 1 (PC1) splits the samples according to MMP-3 genetic status. (**B**) Volcano plot of differentially expressed genes (DEGs) between MMP-3 KO MCAO and WT MCAO samples. Genes downregulated upon MMP-3 deletion in the subacute stroke phase are marked in blue. Genes upregulated upon MMP-3 deletion in the subacute stroke phase are marked in red. Representative DEGs are labeled in black. (**C**–**F**) Enrichment plots and gene expression heatmaps for (**C**) Hallmark Epithelial-Mesenchymal Transition (EMT), (**D**) Hallmark Apoptosis, (**E**) Hallmark Inflammatory Response, and (**F**) Reactome Integrin Cell Surface Interactions gene sets. Row-wise z-scores were computed using transcripts per million (TPM). Core enriched genes of interest in each gene set are labeled in black. MCAO, middle cerebral artery occlusion with reperfusion.

**Figure A1:**
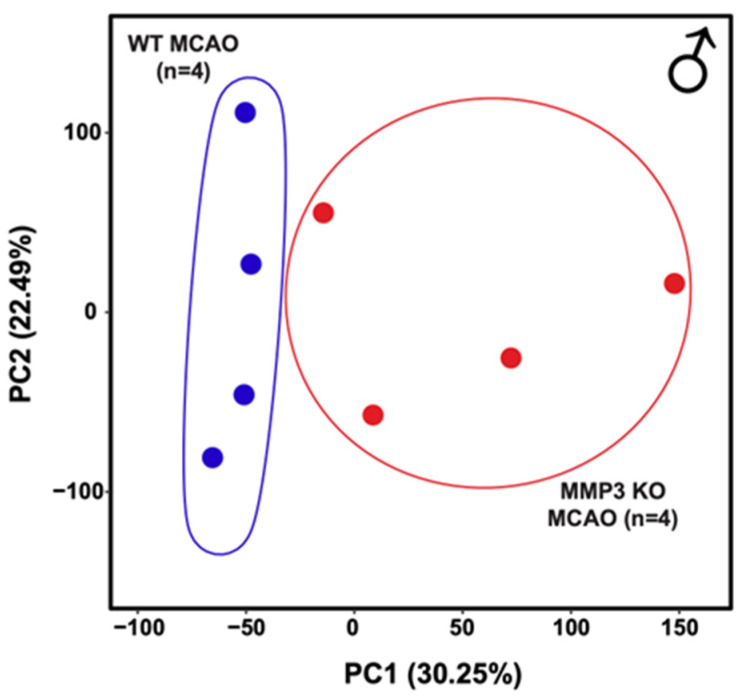
Principal component analysis (PCA) of transcriptomes from whole-brain tissue of male MMP-3 KO (n = 4, red) and male WT (n = 4, blue) mouse brains harvested at 48 h post-stroke. Each dot represents a biological replicate of an RNA-seq sample. Principal component 1 (PC1) splits the samples according to MMP-3 genetic status. MCAO, middle cerebral artery occlusion with reperfusion.

We performed RNA-seq of mouse brain tissue harvested at 48 h post-stroke to investigate the effect of MMP-3 KO on differential gene expression at a global level. Further analysis of differentially expressed genes (DEGs) between MMP-3 KO brains and WT brains was conducted to better understand the molecular mechanisms through which MMP-3 inhibition reduced tissue loss from ischemic stroke. We compared the transcriptomes of male MMP-3 KO mouse stroke brains to those of male WT stroke brains (MMP-3 KO MCAO vs. WT MCAO). Principal component analysis (PCA) showed clear separation between MMP-3 KO and WT stroke brain transcriptomes along PC1 which accounted for the greatest variability (30.25%) in the dataset (Figure 2A and Figure A1). This suggested alterations in gene expression patterns due to MMP-3 deletion. We found 252 downregulated and 81 upregulated genes in MMP-3 KO stroke brains with a fold change >1.5 in either direction (FDR < 0.05) (Figure 2B). The volcano plot (Figure 2B) indicates significant downregulation of genes already implicated in inflammation and apoptotic cell death following stroke such as *Ccr5*, *Casp8*, *Icam2*, *Mmp9*, *Pecam1*, and *Il6*. Further analysis of the TPM values for these genes from WT and MMP-3 KO male stroke brains confirmed our observation from the volcano plot (Figure A4B). These findings suggest that MMP-3 plays a pivotal role in mediating the expression of pathologically relevant genes in the subacute phase of ischemic stroke. 

We further analyzed DEGs to identify enriched or depleted biological pathways in MMP-3 KO mouse stroke brains. Our functional analysis revealed several enriched or depleted pathways in MMP-3 KO stroke brains. Gene Set Enrichment Analysis (GSEA) revealed negative enrichment scores (NES), indicating downregulation of gene sets for inflammation (78 genes, NES= −1.88), epithelial-mesenchymal transition (EMT) (98 genes, NES= −2.26), apoptosis (71 genes, NES= −1.63), and integrin cell surface interactions (40 genes, NES= −2.24) in brains of male MMP-3 KO MCAO mice (Figure 2C–F). This highlights the multifaceted role of MMP-3 in regulating several key biological processes after ischemic insult to the central nervous system (CNS). Notably, the negative enrichment scores across these pathways point to a potential protective or modulatory effect of MMP-3 knockout against ischemia-induced pathological changes. Key EMT-related genes that were downregulated upon MMP-3 KO in male stroke brains included *Fbn1*, *Fbln1*, *Tgfb1*, *Tgfbr3*, *Snai2*, and *Fgf2* (Figure 2C). Male MMP-3 KO stroke brains also exhibited downregulation of apoptosis-related genes such as *Casp9*, *Casp7*, *Bmf*, *Casp1*, *Bid*, *Casp6*, *Casp3*, *Casp2*, *Fas*, *Casp4*, and *Casp8* (Figure 2D). Genes related to inflammation such as *Ccl5*, *Cxcl5*, *Il1a*, *Tnfsf10*, *Nfkb1*, *Tnfrsf1b*, *Ccr7*, *Tnfrsf9*, *Ccl7*, *Il1b*, *Il6*, and *Ccl24* were also downregulated upon MMP-3 KO in male stroke brains (Figure 2E). Similarly, genes involved in integrin cell surface interactions including *Itga10*, *Itgb3*, *Vcam1*, *Itgb1*, *Itgae*, *Itgb7*, *Itga1*, *Itga5*, *Icam1*, *Itgb2*, *Pecam1*, and *Icam2* were depleted in male MMP-3 KO stroke brains (Figure 2F).

**Figure 3:**
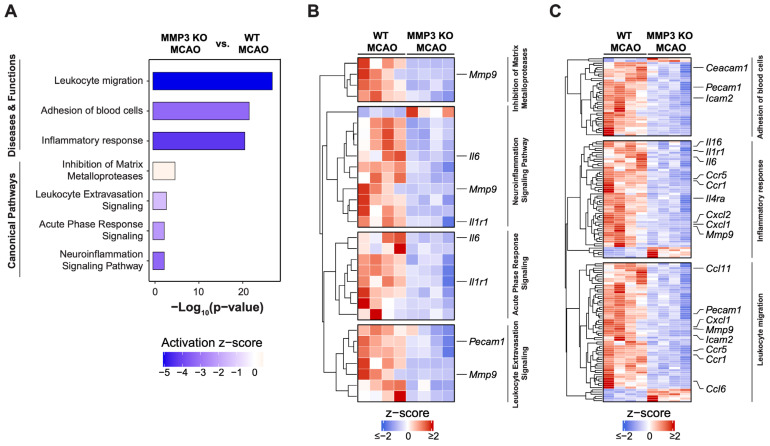
Pathway analysis of global transcriptional changes induced by MMP-3 deletion in male mouse brains 48 h post-stroke. (**A**) Ingenuity Pathway Analysis (IPA) of MMP-3 KO MCAO versus WT MCAO groups reveals pathways affected in male stroke brains. Activation z-scores comparing MMP-3 KO and WT groups are plotted for several signaling pathways and biological functions. Bar graphs show the negative logarithm of the calculated *p*-values for these pathways and functions. (**B**) Heatmap of Canonical Pathway gene expression signatures for leukocyte extravasation, acute phase response, neuroinflammation, and matrix metalloproteases in MMP-3 KO MCAO brains (MMP-3 KO MCAO vs. WT MCAO mice). (**C**) Heatmaps for gene signatures in the Disease and Function Annotation categories for leukocyte migration, blood cell adhesion, and inflammatory response in MMP-3 KO MCAO brains (MMP-3 KO MCAO vs. WT MCAO mice). (Row-wise z-scores were computed using transcripts per million (TPM). Heatmaps illustrate the expression levels (z-scores) of certain genes related to inflammation and cell migration, with color coding indicating the degree of expression from low (blue) to high (red). MCAO, middle cerebral artery occlusion with reperfusion.

We conducted Ingenuity Pathway Analysis (IPA) of DEGs to further interrogate cellular pathways affected by MMP-3 KO in male stroke brains. IPA of canonical pathways revealed downregulation of genes involved in leukocyte extravasation (7 genes, z = −1.414), acute phase response (8 genes, z = −2.236), and neuroinflammation (11 genes, z = −3.317). Meanwhile, MMP-3 KO brains showed enrichment of genes associated with inhibition of matrix metalloproteases (4 genes, z = 0.447) (Figure 3A,B). IPA of the Disease and Function category indicated downregulation of genes involved in leukocyte migration (64 genes, z = −5.066), blood cell adhesion (36 genes, z = −3.231), and the inflammatory response (54 genes, z = −4.305) upon MMP-3 KO in male brains at 48 h post-MCAO (Figure 3A,C). These results further confirm the importance of MMP-3 in regulating expression of pathologically relevant gene networks within the stroke-afflicted brain.

**Figure 4:**
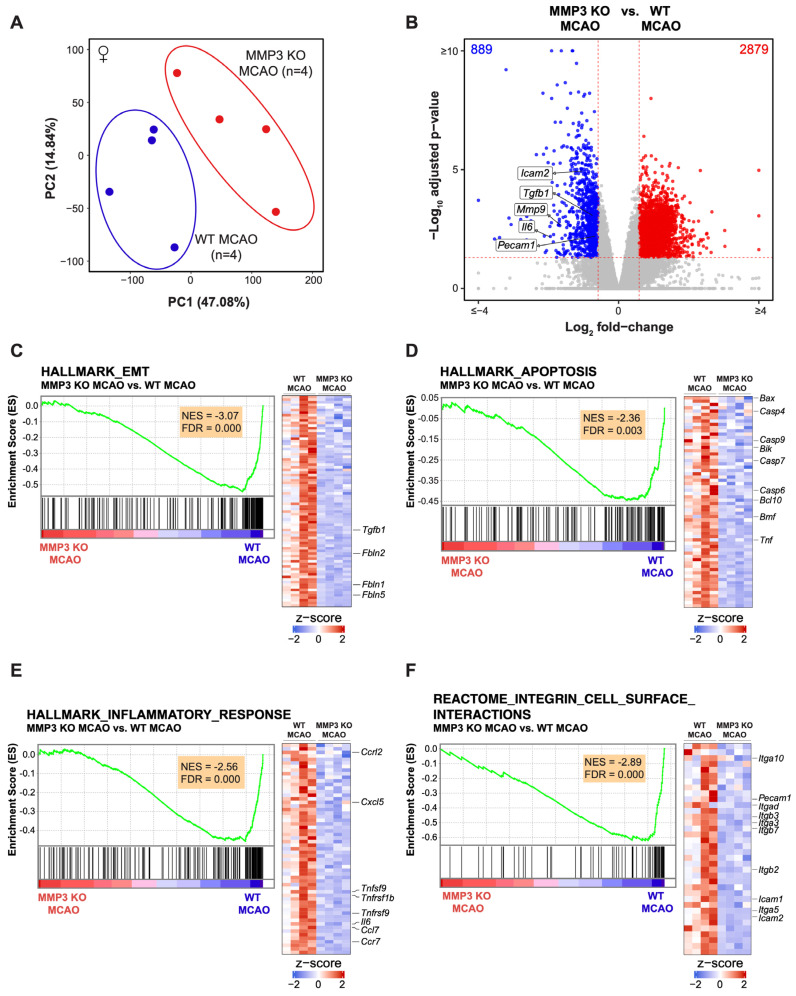
MMP-3 KO induces global transcriptional changes in the brains of female mice by 48 h post-stroke. (**A**) Principal component analysis (PCA) of transcriptomes from whole-brain tissue of female MMP-3 KO (n = 4, red) and female WT (n = 4, blue) mouse brains harvested at 48 h post-stroke. Each dot represents a biological replicate of an RNA-seq sample. Samples were split according to MMP-3 genetic status when plotted along PC1 which accounted for 47.08% of the variance between groups. (**B**) Volcano plot comparing the transcriptomes of MMP-3 KO MCAO versus WT MCAO groups. Statistically significant downregulated DEGs are marked in blue, while statistically significant upregulated DEGs are marked in red. Several representative downregulated genes in MMP-3 KO MCAO brains are labeled in black text. (**C-F**) Enrichment plots and gene expression heatmaps for (**C**) Hallmark Epithelial-Mesenchymal Transition (EMT), (**D**) Hallmark Apoptosis, (**E**) Hallmark Inflammatory Response, and (**F**) Reactome Integrin Cell Surface Interactions gene sets. Row-wise z-scores were computed using transcripts per million (TPM). Core enriched genes of interest in each gene set are labeled in black. MCAO, middle cerebral artery occlusion with reperfusion.

We also compared the transcriptomes of female MMP-3 KO mouse stroke brains (n = 4) to those of female WT stroke brains (n = 4) (MMP-3 KO MCAO vs. WT MCAO). Like male brain samples, PCA was able to clearly separate the transcriptomes of female MMP-3 KO stroke brains from those of female WT stroke brains (Figure 4A). We found 889 downregulated and 2879 upregulated genes in female MMP-3 KO brains when compared to female WT brains at 48 h post-stroke with a fold change >1.5 into either direction (FDR < 0.05) (Figure 4B). Like their male counterparts, brains from MMP-3 KO female mice had downregulated expression of pathologically relevant genes such as *Icam2*, *Tgfb1*, *Mmp9*, *Il6*, and *Pecam1* at 48 h post-MCAO (Figure 4B and Figure A4B).

We performed further analysis of DEGs in female stroke brains to better assess the effects of MMP-3 KO during the subacute stroke phase (Figure 4). GSEA showed negative enrichment scores, indicating depletion, for sets of genes involved in EMT (81 genes, NES = −3.07), apoptosis (64 genes, NES = −2.36), inflammatory response (59 genes, NES = −2.56), and integrin cell surface interactions (36 genes, NES = −2.89) (Figure 4C–F). Key EMT-related genes that were downregulated in female MMP-3 KO stroke brains included *Fbln5*, *Fbln1*, *Fbln2*, and *Tgfb1* (Figure 4C). The apoptosis-related genes *Bax*, *Casp4*, *Casp9*, *Bik*, *Casp7*, *Casp6*, *Bcl10*, *Bmf*, and *Tnf* were all decreased in female MMP-3 KO brains when compared to female WT brains at 48 h post-MCAO (Figure 4D). Furthermore, genes related to the inflammatory response such as *Ccrl2*, *Cxcl5*, *Tnfsf9*, *Tnfrsf1b*, *Tnfrsf9*, *Il6*, *Ccl7*, and *Ccr7*, and genes involved in integrin cell surface interactions including *Itga10*, *Pecam1*, *Itgad*, *Itgb3*, *Itga3*, *Itgb7*, *Itgb2*, *Icam1*, *Itga5*, and *Icam2* were downregulated upon MMP-3 KO in female stroke brains (Figure 4E,F).

**Figure 5:**
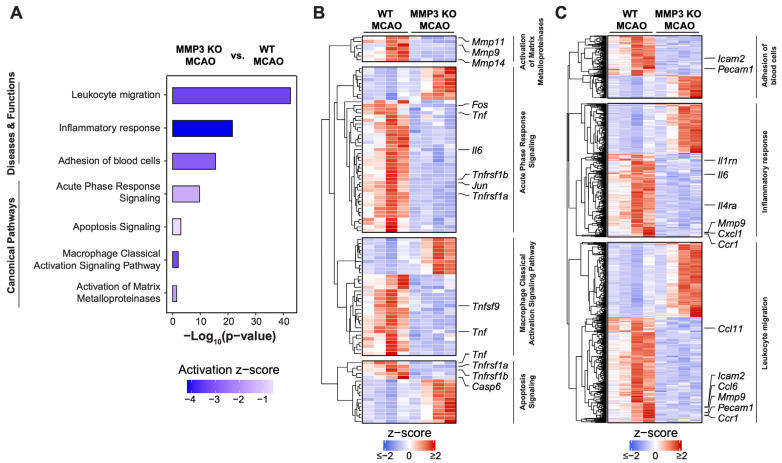
Pathway analysis of global transcriptional changes induced by MMP-3 deletion in female mouse brains at 48 h post-stroke. (**A**) Ingenuity Pathway Analysis (IPA) results of pathways affected by MMP-3 genetic deletion in female stroke brains. Activation z-scores comparing MMP-3 KO and WT groups are plotted for several signaling pathways and biological functions. Bar graphs show the negative logarithm of the calculated *p*-values for these pathways and functions. (**B**) Heatmap of Canonical Pathway gene expression signatures for apoptosis signaling, acute phase response signaling, macrophage classical activation signaling, and activation of matrix metalloproteases in female MMP-3 KO stroke brains (MMP-3 KO MCAO vs. WT MCAO mice). (**C**) Heatmaps for gene signatures in the Disease and Function Annotation categories for leukocyte migration, adhesion of blood cells, and inflammatory response in female MMP-3 KO stroke brains (MMP-3 KO MCAO vs. WT MCAO mice). Row-wise z-scores were computed using transcripts per million (TPM). Heatmaps illustrate the expression levels (z-scores) of certain genes related to inflammation and cell migration, with color coding indicating the degree of expression from low (blue) to high (red). MCAO, middle cerebral artery occlusion with reperfusion.

We performed IPA of transcriptome data from brains of female MMP-3 KO and female WT mice at 48 h post-stroke (Figure 5). Canonical pathway analysis revealed downregulation of macrophage classical activation signaling (32 genes, z = −3.212), acute phase response signaling (45 genes, z = −1.474), apoptosis signaling (17 genes, z = −0.6), and activation of matrix metalloproteases (7 genes, z = −1.667) (Figure 5A,B). Analysis of pathways in the Disease and Function category indicated downregulation of leukocyte migration (436 genes, z = −3.331), adhesion of blood cells (151 genes, z = −2.864), and the inflammatory response (323 genes, z = −4.186) in female MMP-3 KO brains (Figure 5A,C). 

Hierarchical clustering analysis was used to examine differences in expression of specific genes from the canonical pathways and disease and functions (activation z-score) results in females. Female MMP-3 KO stroke brains showed downregulation of the macrophage classical activation signaling genes *Tnf* and *Tnfsf9*, and decreased expression of the acute phage response genes *Tnfrsf1a*, *Jun*, *Tnfrsf1b*, *Il6*, *Tnf*, and *Fos*. Furthermore, female MMP-3 KO stroke brains showed decreased expression of apoptosis signaling genes *Casp6*, *Tnfrsf1b*, *Tnfrsf1a*, and *Tnf*, and downregulated expression of matrix metalloprotease genes *Mmp14*, *Mmp9*, and *Mmp11* (Figure 5B). Similar analysis of genes in the Disease and Function categories indicated that female MMP-3 KO stroke brains had decreased expression of leukocyte migration genes *Ccr1*, *Pecam1*, *Mmp9*, *Ccl6*, *Icam2*, and *Ccl11*. Female MMP-3 KO stroke brains also had decreased expression of blood cell adhesion genes *Pecam1* and *Icam2*, and decreased expression of inflammatory response genes *Ccr1*, *Cxcl1*, *Mmp9*, *Il4ra*, *Il6*, and *Il1rn* (Figure 5A–C).

**Figure 6:**
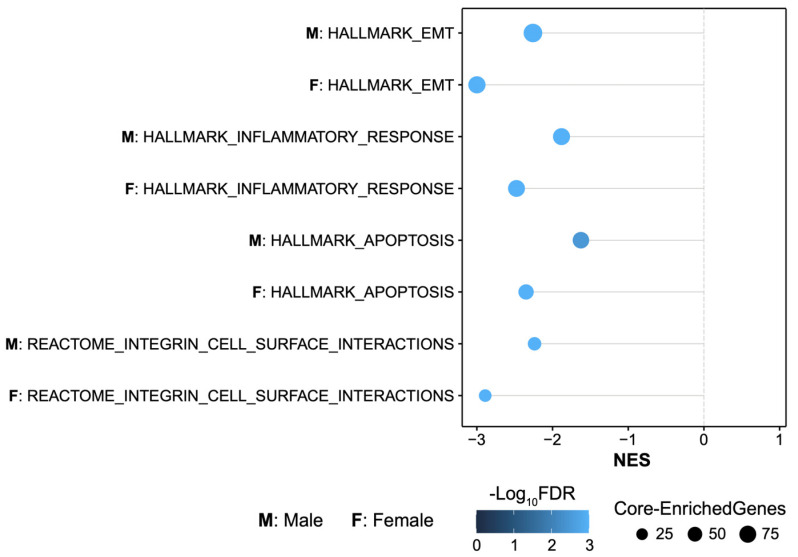
Pathway Enrichment results across key biological processes stratified by sex. Bubble plot of GSEA results in males (M) and females (F) (MMP-3 KO MCAO vs. WT MCAO) across several key biological processes: integrin cell surface interactions, apoptosis, inflammatory response, and epithelial-mesenchymal transition. Normalized enrichment score (NES) is plotted on the *x*-axis. Bubble colors correspond to FDR-q value. The size of the bubbles corresponds with the number of enriched genes in each gene set. MCAO, middle cerebral artery occlusion with reperfusion.

We compared pathway enrichment results between male and female mouse brains to assess potential sex differences in the effects of MMP-3 inhibition on stroke infarct size (Figure 6). Both males (M) and females (F) showed significant negative normalized enrichment scores (NES) across several key biological processes, including integrin cell surface interactions, apoptosis, inflammatory response, and EMT. However, the data showed a greater magnitude of pathway depletion in female MMP-3 KO stroke brains than in male MMP-3 KO stroke brains for all gene sets (Figure 6).

### 2.3. MMP-3 KO Decreases EMT Gene Expression in the Subacute Stroke Phase 

**Figure A2:**
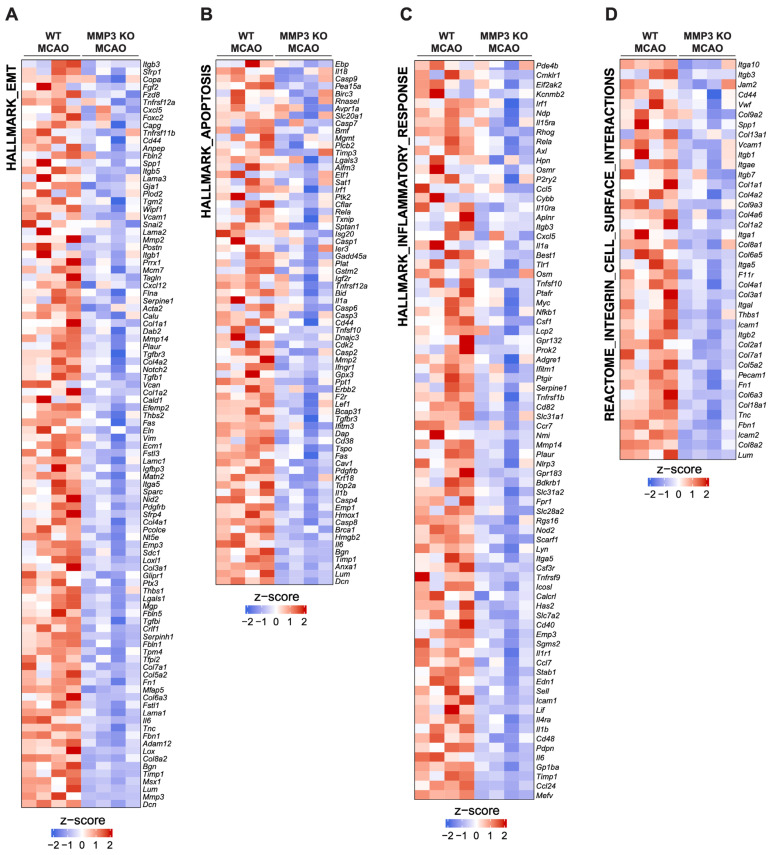
Heatmaps comparing gene expression profiles for (**A**) Hallmark Epithelial-Mesenchymal Transition (EMT), (**B**) Hallmark Apoptosis, (**C**) Hallmark Inflammatory Response, (**D**) Hallmark Integrin Cell Surface Interactions between male MMP-3 KO and male WT MCAO mice. Each panel (**A**–**D**) includes genes not explicitly highlighted in Figure 2. MCAO, middle cerebral artery occlusion with reperfusion. Red color indicates increased expression. Blue color indicates decreased expression.

**Figure A3:**
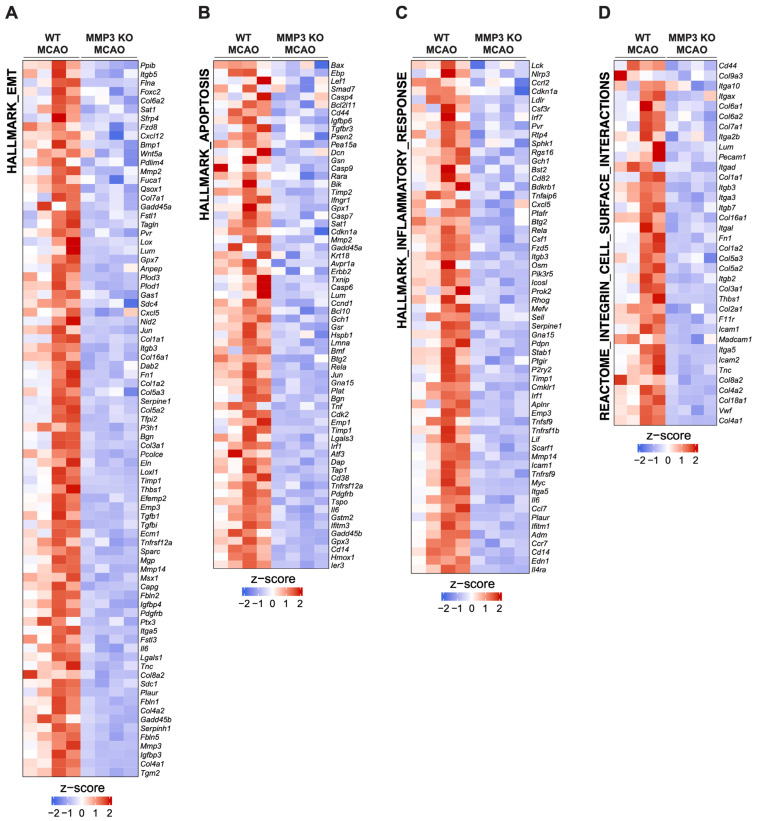
Heatmaps depicting gene expression profiles for the full set of genes in the (**A**) Hallmark Epithelial-Mesenchymal Transition (EMT), (**B**) Hallmark Apoptosis, (**C**) Hallmark Inflammatory Response, (**D**) Hallmark Integrin Cell Surface Interactions sets between female MMP-3 KO and female WT MCAO mice. Each panel (**A**–**D**) includes genes not explicitly highlighted in Figure 4. MCAO, middle cerebral artery occlusion with reperfusion. Red color indicates increased expression. Blue color indicates decreased expression.

To identify possible mechanisms underlying MMP-3 KO’s effect on reducing stroke infarct volume, we performed additional clustering analysis of data from four samples each of MMP-3 KO and WT stroke brains. Clustering clearly distinguished the MMP-3 KO samples from WT samples (Figure 2C, Figure 4C, Figure A2 and Figure A3). 

MMP-3 is a secreted enzyme that degrades components of the extracellular matrix. Although it has important roles in development and tissue remodeling, MMP-3 induction can also be pathogenic. For instance, MMP-3 is upregulated in breast cancer [34], and MMP-3 can induce EMT and promote malignant transformation in cultured cells [35,36,37]. In vascular endothelial cells (ECs), proinflammatory factors can induce a very similar process termed endothelial-mesenchymal transition (EndMT) [38], which destabilizes blood vessels and contributes to cardiovascular disease [39,40]. In fact, ECs are reported to undergo EndMT following tMCAO [41]. Further analysis of DEGs from our RNA-seq data revealed that MMP-3 KO decreased expression of genes related to EMT in male mouse brains during the subacute stroke phase. Specifically, GSEA results showed downregulation of EMT-related genes in males (98 genes, NES = −2.26) (Figure 2C) and in females (81 genes, NES = −3.07) (Figure 4C). Key EMT-related genes that were downregulated in MMP-3 KO males included *Fbn1*, *Fbln1*, *Tgfb1*, *Tgfbr3*, *Snai2*, and *Fgf2*, while female MMP-3 KO stroke brains showed downregulation of *Fbln5*, *Fbln1*, *Fbln2*, and *Tgfb1*. Thus, although both male and female MMP-3 KO mice showed downregulation of EMT-related genes, there were still differences in the expression signatures between sexes. For instance, MMP-3 KO decreased *Snai1* expression to a greater extent in female stroke brains (Log_2_FC = −1.91, FDR = 1.01e−8) than in male stroke brains (Log_2_FC = −0.99, FDR = 0.058). In addition to *Snai1*, expression of the key EndMT/EMT-related genes *Tgfb1* and *Twist1* was decreased to a greater extent in females than in males. Nevertheless, our analyses clearly demonstrate that MMP-3 KO attenuated EndMT- and EMT-related gene expression in the brain during the subacute stroke phase, which correlated with reduced infarct volume. 

### 2.4. MMP-3 KO Attenuates Inflammatory Mediator Gene Expression in the Brain during the Subacute Stroke Phase

Ischemic stroke elicits an inflammatory response that contributes to BBB breakdown and further brain tissue damage [42]. Thus, dampening neuroinflammation is an attractive therapeutic strategy for stroke. In both sexes, MMP-3 KO decreased inflammatory gene signatures in the brain during the subacute stroke phase. When compared to WT stroke brain controls, male MMP-3 KO stroke brains had decreased expression of 78 inflammation-related genes (NES= −1.88) (Figure 2E), while female MMP-3 KO stroke brains showed downregulation of 59 inflammation-related genes (NES= −2.56) (Figure 4E). Our IPA results revealed that female MMP-3 KO stroke brains had negative enrichment scores for inflammation-associated cellular pathways such as leukocyte migration (z = −3.331), inflammatory response (z = −4.186), macrophage classical activation signaling pathway (z = −3.212), and acute phase response signaling (z = −1.474) (Figure 5A,B). Key inflammation-associated genes downregulated upon MMP-3 KO in female stroke brains included *Tnfsf9*, *Il6*, *Tnfrsf9*, *Tnfrsf1b*, *Ccrl2*, *Cxcl5*, *Ccl7*, *Ccr7*, *Fos*, *Tnf*, *Jun*, *Tnfrsf1a*, *Icam2*, *Pecam1*, *Il1rn*, *Il4ra*, *Mmp9*, *Cxcl1*, *Ccr1*, and *Ccl11* (Figure 4E, Figure 5A–C and Figure A3C). IPA of males indicated that MMP-3 KO downregulated cellular pathways for leukocyte migration (z = −5.066), adhesion of blood cells (z = −3.231), inflammatory response (z = −4.305), leukocyte extravasation signaling (z = −1.414), acute phase response signaling (z = −2.236), and neuroinflammation signaling (z = −3.317) (Figure 3A,B). Key inflammation-associated genes downregulated upon MMP-3 KO in male stroke brains included *Ccr1*, *Cxcl1*, *Ccl11*, *Cxcl2*, *Ccl6*, *Il4ra*, *Irf1*, *Il15ra*, *Ccl5*, *Il10ra*, *Itgb3*, *Cxcl5*, *Il1a*, *Tlr*, *Tnfsf10*, *Nfkb1*, *Csf1*, *Adgre1*, *Tnfrsf1b*, *Ccr7*, *Mmp14*, *Mmp9*, *Nlrp3*, *Itga5*, *Tnfrsf9*, *Cd40*, *Il1r1*, *Ccl7*, *Sell*, *Icam1*, *Icam2*, *Lif*, *Il1b*, *Il6*, *Ccl24*, *Pecam1*, and *Ceacam1* (Figure 2E, Figure 3A–C and Figure A2C). Overall, female brains had more extensive downregulation of hallmark inflammation genes in the subacute stroke phase upon MMP-3 KO when compared to their MMP-3 KO male counterparts. In addition, IPA revealed subtle differences in modulation of inflammation- and immune-related gene networks upon MMP-3 KO between males and females; both sexes had depletion of genes in the leukocyte migration, inflammatory response, and acute phase response signaling categories. However, male stroke brains with MMP-3 KO showed downregulation of genes in the neuroinflammation signaling pathway, while female MMP-3 KO stroke brains additionally showed downregulation of genes involved in the macrophage classical activation signaling pathway (Figure 2A–C and Figure 4A–C). Nevertheless, MMP-3 deletion significantly downregulated several inflammatory pathways in both males and females during the subacute stroke phase. 

### 2.5. MMP-3 KO Reduces Apoptotic Gene Expression in the Brain during the Subacute Stroke Phase 

Apoptosis contributes to a significant proportion of neuronal death following acute brain ischemia [43]. Cerebral ischemia triggers two general pathways of apoptosis: the intrinsic pathway and the extrinsic pathway. The intrinsic pathway of apoptosis is triggered by various internal cellular stressors such as nutrient or oxygen depletion from ischemia and results in mitochondrial release of cytochrome c, formation of the apoptosome, and activation of executioner caspase-3 and caspase-7 by initiator caspase-9. On the other hand, the external pathway of apoptosis involves cell surface death receptor signaling that activates initiator caspase-8 to induce downstream executioner caspases [43]. Our GSEA results of male mouse brains revealed depletion of 71 genes related to both extrinsic and intrinsic apoptotic signaling pathways upon MMP-3 KO in the subacute stroke phase (NES = −1.63) (Figure 2D). Males with MMP-3 KO showed decreased gene expression of *Casp9*, *Casp7*, *Bmf*, *Casp1*, *Bid*, *Casp6*, *Casp3*, *Casp2*, *Fas*, *Casp4*, *Casp8*, *Aifm3*, *Irf1*, *Tnfrsf12a*, *Il1a*, *Tnfsf10*, *Ifngr1*, *Tgfbr3*, *Dap*, *Cd38*, *Il6*, and *Anxa1* (Figure 2D and Figure A2B). In female MMP-3 KO mice, GSEA detected negative enrichment or downregulation of 64 genes related to apoptosis in the subacute stroke phase when compared to brains from WT controls (NES = −2.36) (Figure 4D). In addition to GSEA, IPA of RNA-seq data revealed downregulation of apoptosis signaling (z = −0.6) in the canonical pathway category for female MMP-3 KO mice in the subacute phase of stroke (Figure 5A). Representative apoptotic genes that were downregulated in female MMP-3 KO stroke brains included *Bax*, *Casp4*, *Casp9*, *Bik*, *Casp7*, *Casp6*, *Bcl10*, *Bmf*, *Tnf*, *Tnfrsf1a*, and *Tnfrsf1b* (Figure 4D, Figure 5B and Figure A3B). Overall, MMP-3 KO downregulated apoptotic gene expression to a greater extent in females than in males, but still had significant effects in both sexes.

### 2.6. MMP-3 KO Downregulates Expression of Genes Involved in Integrin Cell Surface Interactions

After stroke, the cerebral vasculature experiences altered integrin gene expression and degradation of the surrounding ECM [44]. Integrins are heterodimeric transmembrane proteins formed by the non-covalent binding of α and β subunits which can form 24 known combinations with varying roles [45]. The α subunit determines ligand binding specificity, while both the α and β subunits mediate intracellular signal transduction [46,47]. Upregulation of integrins following stroke is associated with angiogenesis, which plays an important role in tissue repair after stroke. However, this also contributes to BBB dysfunction and can contribute to IR injury in the subacute stroke phase [44]. We analyzed gene sets for integrin cell surface interactions in the mouse M2 REACTOME category to further explore biological mechanisms that might explain reduced infarct volume in MMP-3 KO mice. GSEA revealed that MMP-3 KO downregulated 40 genes related to integrin cell surface interactions in male mice (NES = −2.24) (Figure 2F and Figure A2D). Key genes downregulated in male MMP-3 KO stroke brains included *Itga10*, *Itgb3*, *Vwf*, *Col9a2*, *Col13a1*, *Vcam1*, *Itgb1*, *Itgae*, *Itgb7*, *Col1a1*, *Col4a2*, *Col9a3*, *Col4a6*, *Col1a2*, *Itga1*, *Col8a1*, *Col6a5*, *Itga5*, *Col4a1*, *Col3a1*, *Icam1*, *Itgb2*, *Col2a1*, *Col7a1*, *Col5a2*, *Pecam1*, *Col6a3*, *Col18a1*, *Tnc*, *Icam2*, and *Col8a2* (Figure 2F and Figure A2D). In female mice, MMP-3 genetic deletion decreased the expression of 36 genes related to integrin cell surface interactions at 48 h post-stroke (NES = −2.89) (Figure 4F and Figure A3D). Key genes downregulated in female MMP-3 KO stroke brains included *Pecam1*, *Itgb2*, *Icam1*, *Itgb7*, *Icam2*, *Itga10*, *Itgb3*, *Itgad*, *Itga3*, and *Itga5* (Figure 4F and Figure A3D). Females showed a larger negative enrichment score than males, but MMP-3 KO nevertheless downregulated integrin signaling gene expression signatures in both sexes during the subacute stroke phase, which correlated with reduced infarct volume.

## 3. Discussion

The pathophysiology of ischemic stroke is complex, and damage to the CNS occurs through multiple mechanisms. Therefore, the Stroke Treatment Academic Industry Roundtable (STAIR) recommends targeting multiple pathways for future stroke therapies. Identifying druggable targets that regulate multiple pathophysiological cascades in the early stages of stroke is an attractive therapeutic strategy. 

A large amount of brain damage following stroke is caused by reperfusion injury and BBB breakdown. The BBB plays crucial roles in maintaining brain homeostasis by providing a structural barrier that regulates molecular and cellular trafficking between the brain and circulatory system [48]. Thus, preserving BBB integrity is crucial for improving stroke outcome. Disruption of BBB tight junctions following ischemic stroke results from oxidative stress, EndMT, upregulation of proinflammatory factors, and induction of MMPs [15,49,50]. MMP-3 is a major inducible MMP and can activate latent pro-MMP-9 [28,29,30]. MMP-3 levels sharply increase within several hours after stroke; this coincides with the initial stages of BBB disruption [31]. Studies of human brains report upregulation of both MMP-3 and MMP-9 following stroke [51]. However, data from experiments using MMP-9 KO and MMP-3 KO mice suggest that MMP-3 contributes to delayed tPA-induced intracerebral hemorrhage more than MMP-9 does [31]. Moreover, pharmacological inhibition of MMP-3 improves stroke outcome and decreases hemorrhagic transformation in a diabetic female rat model of stroke [52]. However, the molecular mechanisms through which MMP-3 inhibition improves stroke outcome independent of downstream MMP-9 and MMP-2 activation remain largely unexplored. We hypothesized that inhibition of MMP-3 may improve stroke outcome through several other biochemical and cellular pathways in addition to activation of other latent MMPs. 

We used a well-established transient MCAO/R model to study ischemic stroke [53]. In our study, genetic deletion of MMP-3 in mice reduced infarct volume following MCAO/R. We are the first to use RNA-sequencing analysis to identify global transcripts differentially expressed in MMP-3 KO mouse brains in the subacute stage of stroke. In male MMP-3 stroke brains, RNA-seq analysis identified 252 downregulated and 81 upregulated genes. In female stroke brains, MMP-3 deletion downregulated 889 genes and upregulated 2879 genes. Bioinformatic analysis of whole stroke brain RNA-seq data revealed depletion of gene signatures related to EMT, neuroinflammation, apoptosis, and integrin cell surface interactions upon MMP-3 deletion in both males and females. Here we present the first comprehensive analysis of gene networks in the brain altered by MMP-3 KO that correlate with reduced infarct volume following stroke.

Ischemic stroke triggers intrinsic and extrinsic apoptotic cell death pathways in brain cells, including neurons and endothelial cells (ECs) critical for BBB function. The intrinsic apoptotic pathway is triggered by energy failure from lack of oxygen and glucose, oxidative stress, and sodium and calcium imbalance from glutamate excitotoxicity [43]. During intrinsic apoptosis, cytochrome C is liberated from the mitochondria where it joins with Apaf-1 to activate initiator caspases and executioner caspase-3 and caspase-7 [43]. Stroke also activates apoptosis in injured brain cells through the extrinsic pathway, which occurs through signaling between tumor necrosis factor (TNF), Fas, and Trail cell surface death receptors. These external signals activate effector caspase-8 and caspase-10, which then converge on executioner caspases to propagate the apoptotic cascade [43]. Preclinical studies report that silencing of pro-apoptotic Bcl-2 family members improves neurological outcome in rodent models of stroke [54]. Interestingly, our RNA-seq analysis indicated downregulation of genes for both the intrinsic and extrinsic apoptosis pathways in the brains of male and female MMP-3 KO mice during the subacute stroke phase. Male and female MMP-3 KO brains both showed depletion of executioner *Casp7* as well as other caspases such as *Casp9, Casp4,* and *Casp6.* Male MMP-3 KO brains showed downregulation of the cell membrane death receptor *Fas* and its downstream effector *Bid,* while female MMP-3 KO brains showed downregulation of the TNF signaling genes *Tnf*, *Tnfrsf1a*, and *Tnfrsf1b*. Compared to male brains, female brains had larger negative enrichment scores for the hallmark apoptosis gene set upon genetic ablation of MMP-3. In addition, IPA revealed a significant negative activation z-score for apoptosis signaling genes in female MMP-3 KO brains but not in males. Apoptosis occurs in multiple brain cell types after stroke. Downregulation of intrinsic apoptotic gene expression by MMP-3 KO may reduce infarct size after stroke by limiting IR injury and neuronal loss from intrinsic apoptosis induced by oxygen and glucose deprivation. On the other hand, downregulation of extrinsic apoptosis factor gene expression by MMP-3 KO may reduce stroke infarct volume by blocking apoptosis of neurons and cells of the BBB induced by death receptor signaling during the inflammatory response following the initial ischemic insult. We analyzed bulk RNA from whole brain tissue, so it remains unclear whether MMP-3 KO downregulated genes for apoptosis in neurons and ECs or in other cell types within the brain during the subacute stroke phase. Nevertheless, apoptosis results in a significant amount of tissue loss following stroke, and our results show that MMP-3 inhibition decreases apoptotic signatures and infarct size in the brain post-MCAO. MMP-3 inhibition may directly suppress apoptosis in neurons to reduce infarct volume, or MMP-3 inhibition may reduce infarct volume through downregulation of apoptosis in cell types that form the BBB. Nevertheless, our research suggests that MMP-3 is a pro-apoptotic factor in the stroke-afflicted brain.

Ischemia triggers the release of inflammatory factors from dying cells and stimulates an immune response that damages the BBB and leads to further tissue loss [55]. Damage-associated molecular patterns (DAMPs) and proinflammatory cytokines from injured tissue are released into the circulation and recruit peripheral immune cells such as macrophages, neutrophils, and T cells into the CNS through the compromised BBB [56,57]. DAMPs and cytokines bind to receptors on infiltrating immune cells and cause the upregulation and subsequent secretion of more inflammatory factors such as MMP-9, TNF-α, and interleukin (IL)-1β [56]. Research shows that activation of monocytes after stroke increases NF-κB signaling and TNF-α production, which correlates with worse stroke outcome [58]. Furthermore, incomplete clearance of DAMPs prolongs inflammation and exacerbates neuronal injury in mouse models of ischemic stroke [59]. Therefore, dampening the expression of proinflammatory factors within the brain may limit IR injury following ischemic stroke. GSEA of our transcriptomic data revealed that MMP-3 deletion significantly downregulated expression of genes in the hallmark inflammation gene set during the subacute stroke phase. Furthermore, male and female MMP-3 KO brains had negative activation z-scores for gene signatures indicative of acute phase response (APR) signaling, neuroinflammation, and the inflammatory response. The acute phase response is the early inflammatory response to infection or tissue injury such as stroke [60]. Clinical studies show that the magnitude of the acute phase response is strongly correlated with infarct size and long-term recovery in stroke patients; patients with higher serum APR protein levels often have poor neurological outcome [61]. Hierarchical clustering analysis of differentially expressed genes (DEGs) in MMP-3 KO mouse stroke brains revealed decreased expression of genes encoding TNF receptors and proinflammatory cytokines/chemokines and their receptors in both sexes. For example, MMP-3 KO downregulated brain expression of TNF receptor genes *Tnfrsf10b*, *Tnfrsf12a*, *Tnfrsf1b*, and *Tnfrsf1a*. Proinflammatory cytokines such as Il-6 are increased following stroke and correlate with poor stroke outcome [62]. Furthermore, chemokines secreted by activated microglia and astrocytes following stroke attract immune cells to the site of damage [49]. In addition to *Il6,* we also observed downregulation of *Il1a* and *Il1b* in male stroke brains, and downregulation of *Il1r1* in female stroke brains upon genetic ablation of MMP-3. Chemokines such as *Cxcl5* and *Ccr7* were also downregulated in both male and female stroke brains upon MMP-3 KO. We observed a sex-specific downregulation of other chemokines and *Nfkb1* upon MMP-3 deletion, but this was unsurprising considering well-established sex differences in the inflammatory response to stroke [63]. Nevertheless, our analysis suggests that MMP-3 inhibition may decrease stroke infarct volume by dampening the expression of inflammatory mediators that attract peripheral immune cells to the ischemic lesion and potentiate IR injury.

IR injury promotes inflammation and BBB disruption that enables invasion of peripheral immune cells into the brain [64]. Circulating lymphocytes contact inflamed vessels after stroke via P-selectin glycoprotein ligand-1, which interacts with E-selectin and P-selectin on ECs of the BBB [65]. Integrins on lymphocytes then bind to vascular cell adhesion molecule 1 (VCAM-1) and intercellular adhesion molecule 1 (ICAM-1) on ECs, which allows for leukocyte adhesion and migration along the BBB [66]. In addition, interactions between PECAM-1 and CD99 on leukocytes and ECs disrupt tight junctions and EC structure to promote diapedesis [67]. Preclinical studies indicate that trafficking of leukocytes into the brain after stroke worsens outcomes due to neuroinflammation-mediated neuronal cell death [68]. Integrin receptor α5β1 has been reported to promote leukocyte infiltration and BBB dysfunction; mice with conditional EC knockout of α5 integrin (α5 KO) show smaller infarct size and other markers of improved outcome in early stroke [69]. Integrin signaling also plays an important role in BBB homeostasis and becomes perturbed after ischemic stroke. For instance, upregulation of αvβ3 levels post-stroke induces the internalization of important tight junction proteins occludin and zonula occludens (ZO-1) in ECs and promotes the secretion of MMP-2 and MMP-9 [70,71,72]. Therefore, downregulation of integrins and adhesion molecules that compromise BBB integrity or facilitate the migration and invasion of peripheral immune cells into the CNS following stroke may limit IR injury. In both male and female stroke brains, we found that MMP-3 KO decreased the expression of *Itga10* and *Itgb3*, which encode the subunits that form integrin αvβ3. Thus, MMP-3 inhibition may reduce stroke infarct volume by decreasing the expression of genes that induce BBB dysfunction. In addition, our IPA results showed that MMP-3 KO reduced gene expression related to adhesion of blood cells, integrin signaling, leukocyte migration, and leukocyte extravasation at 48 h post-stroke. Clustering analysis of DEGs in male and female stroke brains revealed that MMP-3 KO decreased expression of genes that belong to the adhesion and leukocyte extravasation pathway such as *Esam*, *Icam1*, *Icam2*, *Mcam1*, and *Pecam1*. Thus, MMP-3 inhibition may reduce infarct volume after stroke by decreasing the expression of genes required for leukocyte adhesion and extravasation through the BBB, thereby limiting IR injury caused by prolonged cytotoxic inflammation. More research is needed to determine what specific cell types in the brain exhibit downregulation of integrins and adhesion molecules in response to MMP-3 inhibition.

In healthy brain blood vessels, specialized microvascular ECs of the BBB form tight junctions and regulate the passage of cells and substances into and out of the CNS. However, ischemic stroke induces endothelial dysfunction and BBB permeability. Epithelial- and endothelial-mesenchymal transition (EMT/EndMT) are pathological processes in which epithelial cells and ECs de-differentiate into mesenchymal cells and lose their specialized function in the vasculature. Although EndMT has important roles in embryogenesis and development, inappropriate activation of EndMT contributes to adult cardiovascular diseases, including stroke [73]. In general, EndMT is characterized by reduced expression of endothelial genes and increased expression of mesenchymal genes associated with transcription factors such as *Twist*, *Smad3*, *Zeb2*, *Snai1*, and *Snai2*. It has been reported that IR injury of the brain coincides with EndMT and vascular fibrosis through activation of the Let-7i/TGF-βR1 double-negative feedback loop [41]. Specifically, induction of TGF-βR1 and downregulation of Let-7i following ischemia modulates inflammatory pathways in ECs, while increasing Let-7i with a TGF-βR1 antagonist reduced infarct volume and BBB permeability and improved neurological outcome [41]. A previous study also shows that the circular RNA DLGAP4 improves stroke outcome by acting as a miR-143 sponge. This increased the expression of tight junction proteins and decreased the expression of mesenchymal genes in brain ECs [74]. Other studies implicate EndMT in BBB dysfunction in neurological diseases other than stroke. For instance, stimulation of human brain ECs with TGF-β1 and IL-1β promoted EndMT and increased markers of multiple sclerosis in vitro. Immunohistochemistry results from the same study also showed EndMT-associated alterations in the brain vasculature of post-mortem multiple sclerosis patient brain tissue [40]. Targeting EndMT may hold therapeutic promise for stroke and other neurological disorders by preserving the structure and function of the BBB. Our RNA-seq analysis showed that genetic ablation of MMP-3 in our mouse model of stroke had a significant effect on EMT/EndMT gene expression in the brain during the subacute stroke phase when BBB dysfunction starts. Both male and female MMP-3 KO stroke brains showed decreased expression of genes involved in fibulin signaling (*Fbln1*, *Fbn1*, *Fbln2*, *Fbln5*), which are known to induce EMT during embryonic development [75]. Both sexes also exhibited downregulation of *Tgfb1*, which encodes the ligand for TGF-βR1. We also observed sex-specific alterations in other EndMT-associated genes; male MMP-3 KO stroke brains showed downregulation of *Tgfbr3* and the major EMT-associated transcription factor *Snai2*. Overall, our results suggest that MMP-3 inhibition may benefit stroke outcome by reducing expression of transforming growth factor and fibulin signaling factors within the brain to inhibit EndMT. This, in turn, would preserve BBB EC function and limit BBB breakdown and subsequent IR injury to reduce infarct size during the subacute stroke phase. However, since we employed bulk RNA-seq, it is unclear whether MMP-3 KO modulated expression of EndMT-related genes in BBB ECs or other cell types in the brain. Future studies would benefit from using targeted epigenetics assays such as single cell RNA-seq (scRNA-seq) or chromatin immunoprecipitation-seq (ChIP-seq) using brain ECs to see if MMP-3 inhibition does indeed modulate expression of these genes in the endothelium or in other brain cell types following stroke. 

**Figure A4:**
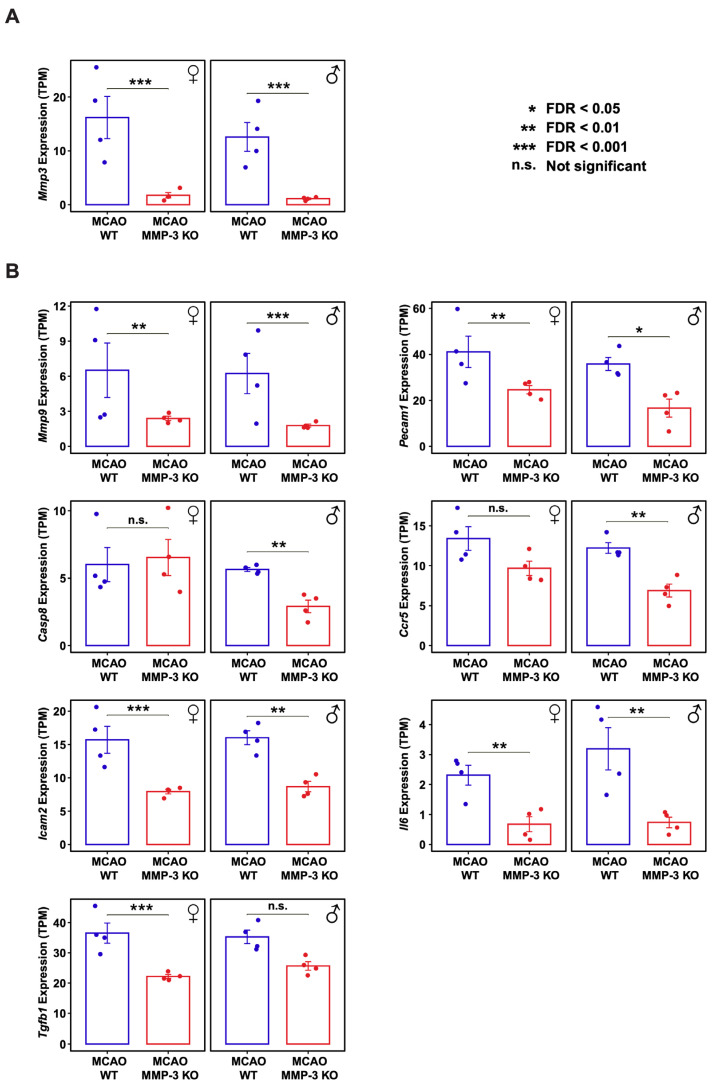
TPM levels of inflammatory mediators from stroke mouse brains harvested at 48 h post-stroke. (**A**) MMP-3 TPM values are higher in the WT MCAO group (blue) than in the MMP-3 KO MCAO group (red). There were no detectable levels of MMP-3 in sham control animals. Following stroke, the MMP-3 expression is significantly induced in WT MCAO group. As anticipated, MMP-3 expression is lower in the MMP-3 KO MCAO group at 48 h post-stroke as shown by RNA-seq analysis. *** FDR < 0.001. (n = 4, WT MCAO; n = 4, MMP-3 KO MCAO). (**B**) Expression of inflammatory mediators at 48 h post-stroke is lower in the MMP-3 KO MCAO group (red) than in the WT MCAO group (blue). * FDR < 0.05; ** FDR < 0.01; *** FDR < 0.001; n.s., not significant (n = 4, WT MCAO; n = 4, MMP-3 KO MCAO). Each dot represents a biological replicate of an RNA-seq sample. MCAO, middle cerebral artery occlusion with reperfusion; TPM, Transcripts Per Million.

Although we observed similar trends in depleted pathways between male and female MMP-3 KO stroke brains, females overall showed a more profound downregulation of gene signatures as reflected in their more negative z-scores and enrichment scores. We observed slightly higher MMP-3 TPM values in female wild-type stroke brains compared to male wild-type stroke brains (Figure A4A). This suggests that females may have greater induction of MMP-3 expression in the brain following stroke than males do. It is possible that estrogen receptor and androgen receptor signaling drive differential expression of MMP-3 between females and males. Future studies should also investigate whether MMP-3 interacts directly or indirectly with estrogen receptors and androgen receptors in the nucleus to regulate gene expression in the brain; this may also account for some of our observed sex differences in response to MMP-3 KO following stroke. Although it is beyond of the scope of our study, future studies should consider age as a biological variable and potential confounder.

Our study had some limitations that can be addressed by future research. We demonstrated that MMP-3 KO reduces cerebral infarct size and is accompanied by global transcriptional changes. Although investigating the long-term neurological outcomes of MMP-3 KO is beyond the scope of this study, it would be beneficial for future research.

In this study, we analyzed bulk RNA harvested from whole brains to assess the effect of MMP-3 KO on stroke pathophysiology. Many cell types within the brain such as neurons, astrocytes, microglia, ECs, vascular smooth muscle cells, neutrophils, macrophages, and T cells, are affected by ischemic stroke in different ways. Gene expression is also context-dependent and determined by the input of many different signals. It is unlikely that MMP-3 regulates the genes identified in our study to the same degree across all cell types in the brain following stroke. Therefore, future studies would benefit from the use of scRNA-seq and other spatial transcriptomics techniques to better identify genes that MMP-3 regulates in specific cell types within the brain. In addition, epigenetic profiling technologies, such as ChIP-seq, should be employed in future research to determine if MMP-3 directly regulates gene expression by acting as a transcription factor or through other indirect mechanisms. The use of an additional genetically engineered mouse model that expresses a catalytically dead MMP-3 mutant may be able to delineate whether the protease activity of MMP-3 is required for its role in regulating gene expression.

In conclusion, genetic deletion of MMP-3 reduced brain infarct volume in our mouse model of ischemic stroke. MMP-3 KO altered gene expression signatures for neuroinflammation, apoptosis, EndMT, and integrin signaling during the subacute stroke phase, which coincides with BBB breakdown and IR injury. MMP-3′s canonical role is proteolytic cleavage of ECM proteins and other secreted extracellular substrates such as latent pro-MMP-9. However, some studies report that MMPs can localize within the nucleus of mammalian cells and exert transcription factor functions to regulate gene expression. For instance, MMP-3 is reported to be a trans regulator of the connective tissue growth factor gene (CCN2/CTGF) in chondrocytes [76]. More research is needed to determine whether MMP-3 directly regulates the expression of genes related to neuroinflammation, apoptosis, and EndMT in brain cells as a transcription factor or through another biochemical pathway such as signaling through extracellular substrate cleavage. Overall, our results highlight MMP-3 as an attractive therapeutic target to improve stroke outcome and our study warrants further investigation of MMP-3′s role in stroke pathophysiology.

## 4. Materials and Methods

### 4.1. Animals

MMP-3 KO mice and littermate controls (8–12 weeks) were obtained from Taconic Biosciences (Rensselaer, NY, USA). Mice were kept at 18–22 °C on a 12 h light-dark cycle. Mice were provided ad libitum access to water and food. 

### 4.2. Animal Model of Stroke

The use of animals for this study was reviewed and approved by Tulane University (New Orleans, LA, USA) and University of California, Riverside (Riverside, CA, USA) Institutional Animal Care and Use Committees. Animals were managed and treated in compliance with the guidelines of Tulane University and UCR animal protocols, the American Veterinary Medical Association, and the National Institutes of Health Guide for the Care and Use of Laboratory Animals.

We used a well-established mouse model of transient focal cerebral ischemia [53]. Stroke surgery was performed as previously published [77,78,79]. Briefly, a 6-0 nylon monofilament (Doccol Corporation, Sharon, MA, USA) was used to induce middle cerebral artery occlusion (MCAO) for 1 h and then removed to enable reperfusion. As a control for the surgical procedure (sham control), mice underwent insertion and immediate removal of the filament. To confirm successful MCAO, we assessed regional cerebral blood flow (rCBF) with a transcranial laser Doppler (Perimed). A rCBF reduction of >80% indicated successful occlusion. Restoration of blood flow to >90% of baseline rCBF indicated successful post-MCAO/R recovery. 

### 4.3. Quantification of Infarct Volume

Triphenyl tetrazolium chloride (TTC, Sigma, St. Louis, MO, USA) staining of mouse brain slices was utilized to evaluate ischemic lesions. To begin, 48 h after MCAO, 1-mm coronal brain sections were incubated in 2% TTC solution as previously published [77,78,79]. Image J software Version 1.54j (National Institutes of Health, Bethesda, MD, USA) was used to measure infarct area. To compensate for edema, cerebral infarct volume was calculated as a percent volume of the contralateral hemisphere: [volume of contralateral hemisphere—(volume of total ipsilesional hemisphere—volume of infarct area)]/volume of contralateral hemisphere. 

### 4.4. RNA Sequencing (RNA-seq)

Poly(A) RNA was isolated using the NEBNext^®^ Poly(A) mRNA Magnetic Isolation Module. RNA-sequencing libraries were constructed using the NEBNext^®^ Ultra™ Directional RNA Library Prep Kit for Illumina^®^ (NEB, Ipswich, MA, USA). Libraries were pooled and sequenced as single-end 75bp on an Illumina NextSeq 500 sequencer with a sequencing depth of 18–30 million reads.

#### 4.4.1. RNA-seq Data Processing

Poly(A), Poly(T), and Illumina Truseq Adapter sequences were trimmed from raw sequencing reads with Cutadapt v2.3. Trimmed reads were aligned to mouse genome version 38 (mm10) using STAR aligner v2.7.0d_0221 [80] with parameters according to ENCODE long RNA-seq pipeline (https://github.com/ENCODE-DCC/long-rna-seq-pipeline, accessed on 10 June 2024). Estimated counts and transcripts per million (TPM) for each gene were quantified by RSEM v1.3.1 [81]. 

#### 4.4.2. RNA-seq Quality Control and Quality Assurance (QC/QA) 

RNA-seq alignment and quantification quality were assessed by FastQC v0.11.5 (https://www.bioinformatics.babraham.ac.uk/projects/fastqc/; RRID: SCR_014583, accessed on 10 June 2024) and MultiQC v1.8 [82]. Biological replicate concordance was assessed by principal component analysis (PCA) and pair-wise Pearson correlation analysis. Lowly expressed genes were filtered out by applying the following criterion: estimated counts (from RSEM) ≥ number of samples * 5. 

#### 4.4.3. Differential Gene Expression and Ingenuity Pathway Analysis (IPA)

Filtered estimated read counts from RSEM were compared with the R Bioconductor package DESeq2 v1.22.2 based on the generalized linear model and negative binomial distribution [83]. Significant differentially expressed genes (DEGs) were identified with the Wald test (BH adjusted *p*-value < 0.05) and had a fold-change >1.5 in either direction. IPA (Qiagen, Santa Clarita, CA, USA) of RNA-seq data was used to further assess cellular pathways affected by MMP-3 deletion in mouse stroke brains. Bioinformatics analysis was performed in the Sanford Burnham Prebys Medical Discovery Institute (SBP) Bioinformatics Core.

#### 4.4.4. Gene Set Enrichment Analysis (GSEA)

Gene set enrichment analysis of MMP-3 KO and MMP-3 WT stroke brain transcriptomic data was performed using the desktop GSEA app version 4.3.2 [84] and TPM values from the RNA-seq samples. GSEA was run using the “Run GSEA” option with default parameters except “Permutation type = gene_set”. The mouse-ortholog hallmark and M2 curated gene sets were used for testing.

### 4.5. Statistical Analysis 

GraphPad Prism, version 6.0, SPSS Version 19.0 and R statistical software version 4.4.1 were used to perform analyses. Unless stated otherwise, one-way ANOVA with Fisher’s LSD post-hoc test was used to assess differences between multiple groups. Results were considered statistically significant at *p* < 0.05. Data are presented as mean ± SEM. 

## Data Availability

The raw data supporting the conclusions of this article will be made available by the authors on request.

## Abbreviations

APR: acute phase response, BBB: blood-brain barrier, DEGs: differentially expressed genes, EC: endothelial cell, ECM: extracellular matrix, EndMT: endothelial-mesenchymal transition, EMT: epithelial-mesenchymal transition, GSEA: Gene Set Enrichment Analysis, HT: hemorrhagic transformation, IPA: Ingenuity Pathway Analysis, IR: ischemia followed by reperfusion, KO: knockout, MCAO/R: middle cerebral artery occlusion/reperfusion, MMP: matrix metalloprotease, NES: negative enrichment score, PCA: Principal Component Analysis.
